# Reporting vital parameters upon referral of patients to the emergency department needs to be improved

**DOI:** 10.1186/1757-7241-21-S2-A44

**Published:** 2013-09-09

**Authors:** Christina Bach, Thomas A Schmidt

**Affiliations:** 1 The Emergency Department, Holbaek University Hospital, Denmark

## Background

The purpose of triage is to prioritize patients and identify those who need immediate attention. Primary triage is attempted by the coordinating physician at the Emergency Department (ED) based primarily on the patient’s vital parameters (blood pressure, heart rate, oxygen saturation, respiration rate and body temperature) received by phone from the referring party, i.e. paramedics or general practitioners (GPs). Secondary triage of the patients is performed minutes after arrival to the ED by nurse or physician. Based on symptoms or by observations of vital parameters, patients are allocated to four different triage categories according to severity. Patients referred by paramedics are subject to basic observations of vital parameters that allow primary triage and subsequent ED preparedness. In this study we evaluated whether the information obtained in the conversation with GPs was sufficient to perform meaningful primary triage. Further, we wanted to elucidate the correlation between primary and secondary triage.

## Methods

The study was a double-blinded prospective observational study. The triage-information cards from patients referred from GPs during daytime were obtained over five days randomly selected over five weeks (all weekdays were represented).

## Results

A total of 50 patients were included. Out of these primary triage was attempted / deemed meaningful in merely 38% (19/50) of the cases. In only 18% (9/50) of the cases any vital parameters were reported from the GP, and for none of the patients admitted all vital parameters were reported. Of the admitted patients with vital parameters reported, only in 44% (4/9) of the cases primary triage was attempted. Of all patients admitted merely 32% (16/50) received both primary and secondary triage. Of these 81% (12/16) were subjected to the same severity category in both triage rounds.

## Conclusion

The information received from GPs referring patients to the ED is limited with regard to vital parameters, and not sufficient to conduct primary triage. The results indicate that when primary triage is performed, it correlates well with secondary triage. Collaboration between referring physicians and the ED should be improved with regard to reporting the patient’s vital parameters.

